# Potential Contribution of Exosomes to the Prion-Like Propagation of Lesions in Alzheimer’s Disease

**DOI:** 10.3389/fphys.2012.00229

**Published:** 2012-07-05

**Authors:** Valérie Vingtdeux, Nicolas Sergeant, Luc Buée

**Affiliations:** ^1^Litwin-Zucker Research Center for the Study of Alzheimer’s Disease, The Feinstein Institute for Medical Research, North Shore-Long Island JewishManhasset, NY, USA; ^2^UMR 837, Institute National pour la Santé et la Recherche Medical, Alzheimer and TauopathiesLille, France; ^3^Faculty of Medicine, Jean-Pierre Aubert Research Centre, Institute of Predictive Medicine and Therapeutic Research, Université Lille Nord de France, UDSL, Place de VerdunLille, France

**Keywords:** Alzheimer’s disease, tauopathies, multivesicular bodies, exosomes, amyloid precursor protein, microtubule-associated tau protein

## Abstract

Since the discovery of prion diseases, the concept has emerged that a protein could be a transmissible pathogen. As such, this transmissible pathogen agent can transfer its pathological mis-folded shape to the same but normally folded protein thus leading to the propagation of a disease. This idea is now extrapolated to several neurological diseases associated with protein mis-folding and aggregation, such as Alzheimer’s disease (AD). AD is a slowly developing dementing disease characterized by the coexistence of two types of lesions: the parenchymal amyloid deposits and the intraneuronal neurofibrillary tangles (NFT). Amyloid deposits are composed of amyloid-beta peptides that derive from sequential cleavages of its precursor named amyloid protein precursor. NFT are characterized by intraneuronal aggregation of abnormally modified microtubule-associated Tau proteins. A synergistic relationship between the two lesions may trigger the progression of the disease. Thus, starting in the medial temporal lobe and slowly progressing through temporal, frontal, parietal, and occipital cortex, the spreading of NFT is well correlated with clinical expression of the disease and likely follows cortico-cortical neuronal circuitry. However, little is known about the mechanism driving the spatiotemporal propagation of these lesions ultimately leading to the disease. A growing number of studies suggest that amyloid deposits and NFT are resulting from a prion-like spreading. In the present chapter, we will develop the current hypotheses regarding the molecular and cellular mechanisms driving the development and spreading of AD lesions from the window of multivesicular endosomes/bodies and exosomes.

## Alzheimer’s Disease, Amyloid Deposits, and APP Metabolism

Alzheimer’s disease (AD) is a slow and progressive disease affecting the brain and characterized by the loss of superior cognitive functions ultimately leading to dementia and death. Two neuropathological brain lesions are found in the brain and their presence is necessary for providing a definite diagnosis of the disease, as firstly described by Alzheimer (1911).

**Figure 1:**
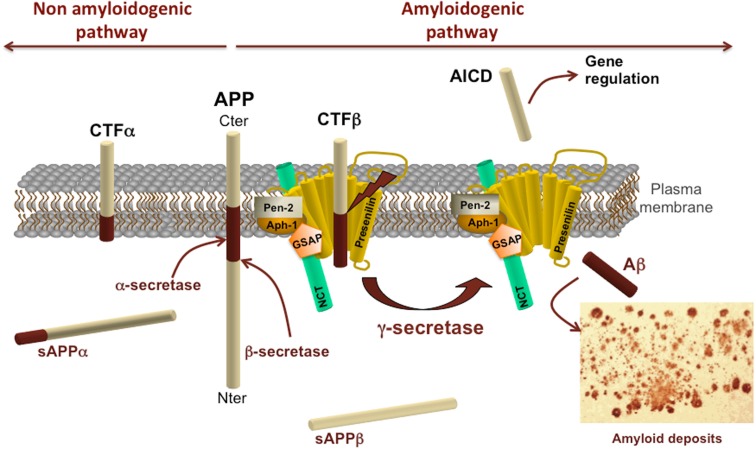
**Amyloid protein precursor structure and metabolism**. Schematic representation of APP processing by α-, β-, and γ-secretases. APP processing by secretase activities is divided into the non-amyloidogenic pathway on the left and the amyloidogenic pathway on the right. α- and β-secretase activities cleave APP in its extracellular domain to release respectively a soluble fragment sAPPα or sAPPβ in the extracellular space and generate carboxy-terminal fragments CTFα or CTFβ. These CTFs can subsequently be processed by γ-secretase complex to generate AICD and Aβ. The γ-secretase complex is composed of presenilin, nicastrin (NCT), γ-secretase activating protein (GSAP), pen-2, and aph-1.

Amyloid deposits are amorphous parenchymal deposits of β-sheet ordered proteinaceous material. They are observed with aging, in AD, Down’s syndrome, dementia with Lewy bodies, and vascular dementia, all of which are aged-related neurodegenerative disorders. The major component of amyloid deposits is a small peptide of 39 to 43 amino acid residues, named Aβ for amyloid-beta peptide (Glenner and Wong, 1984). It derives from a sequence of successive cleavages of a larger precursor protein named APP. *APP* gene is located on the long arm of chromosome 21 at position 21q11.2 (Goldgaber et al., 1987; Kang et al., 1987). APP is a type I transmembrane protein with a large extra amino-terminal membrane domain, a transmembrane domain, and a short carboxy-terminal cytosolic tail composed of 59 amino acids (Figure 1). The principal role of APP remains elusive but several functions are proposed, for instance APP was recently suggested to contribute to iron cellular homeostasis (Duce et al., 2010), to regulate intracellular transport via its interaction with motor proteins such as kinesin, to be a cell surface receptor. Extracellular fragments derived from the cleavage of APP were suggested to be neuroprotective or to promote axon outgrowth (Chasseigneaux et al., 2011) whereas others functions are associated to an ancestral immunological mechanism of defense and would potentially have antibacterial peptide property (Soscia et al., 2011). However, the full spectrum of APP isoform functions remains to be elucidated.

Proteolytic cleavage of APP brings into play sequential events involving first the release of its ectodomain either by α- or β-secretase activities (Figure 1). These cleavages generate carboxy-terminal fragments remaining anchored to the plasma membrane and they shed extracellular soluble fragments, both of which are playing a role in axon outgrowth *in vitro* (Chasseigneaux et al., 2011). APP cleavage by α-secretase generates a soluble APP fragment α (sAPPα) and a carboxy-terminal α fragment composed of 83 amino acids (named C83 or CTFα; for review see Vingtdeux and Marambaud, 2012). This cleavage takes place within the sequence of Aβ peptide thus precluding its formation. This pathway is therefore referred to as the non-amyloidogenic pathway. The α-secretase activity is carried by metalloproteases called A Disintegrin And Metalloprotease (ADAMs). Several ADAM proteases with an α-secretase activity have been identified, including ADAM-17 or TNF-α converting enzyme (TACE; EC 3.4.24.86, peptidase family M12; Buxbaum et al., 1998), ADAM-10 (EC 3.4.24.81, peptidase family M12; (Lammich et al., 1999; Lopez-Perez et al., 2001), and ADAM-9 (EC 3.4.24.; Koike et al., 1999; Hotoda et al., 2002).

The β-secretase cleaves APP at the first amino acid residue of Aβ sequence. The β-cleavage generates a soluble fragment sAPPβ and a CTF comprised of 99 amino acids (C99 or CTFβ). All APP-CTFs (CTFα, β′, and β) can subsequently be cleaved at the juxtamembrane region by the γ-secretase (Figure 1). However, ectodomain cleavage of APP is mandatory to intramembrane γ-secretase proteolysis of APP-CTFs. The APP intracellular domain (AICD or C51) is released from both CTFα and CTFβ by the γ-secretase following the cleavage at the ε-site. However, CTFs can also be processed at the γ-sites but yet AICD of 57 or 59 amino acids have not been detected (for review see Pardossi-Piquard and Checler, 2011). The γ-secretase cleavage of CTFβ represents the last step of Aβ production and is currently considered to be the pathway releasing AICD in the cytoplasm, thus having a potential gene regulatory function together with Fe65 and Tip60 (Konietzko et al., 2010). Following cleavages sites are the γ-sites which produce Aβ species of 43, 42, 40, 39, 38, 37 amino acid long following the rule of tri- or tetrapeptide release (Takami et al., 2009; for review see Karran et al., 2011). The γ-secretase is a multiprotein complex composed of at least four proteins, Presenilin, Pen-2, Aph-1, Nicastrin, and one molecule of each is necessary and sufficient to form an active enzymatic complex (Edbauer et al., 2003; Kimberly et al., 2003; Takasugi et al., 2003; Sato et al., 2007).

The α- and β-secretases are sheddases releasing the extracellular domain of APP as well as several others type I transmembrane proteins. The cleavage and localization of enzyme activity is supposed to occur at the plasma membrane or in early endosomes. As for instance, BACE-1 resides within endosomes and APP endocytosis is a prerequisite for cleavage of APP by BACE-1 and generation of Aβ (Vassar et al., 1999; Walter et al., 2001; Ehehalt et al., 2003). BACE-1 optimal protease activity necessitates an acidic pH and acidification of endosome occurs during the route of endosomes to fuse with lysosomes where BACE-1 is degraded (Koh et al., 2005). Cleavage of APP-CTFs by γ-secretase can occur at several places in the cell (e.g., plasma membrane, endosomes…). Discrepancies exist regarding the cell localization of γ-secretase by-products. Several APP metabolites including APP, APP-CTFs, Aβ, and AICD have been shown to accumulate in multivesicular bodies (MVBs) following treatment of cells with alkalizing drugs (Verbeek et al., 2002; Vingtdeux et al., 2007b). Interestingly and similarly to the effect of Gleevec, alkalizing drugs such as chloroquine, ammonium chloride, bafilomycin A1, block Aβ production without affecting AICD generation (Vingtdeux et al., 2007a). More interestingly, following treatment, the AICD amount raise and AICD is also released outside the cell. Inside the cell AICD is reaching the nucleus (Goodger et al., 2009) where it regulates gene expression such as neprelysin (Pardossi-Piquard et al., 2005; for review see Pardossi-Piquard and Checler, 2011). Interestingly, intracellular AICD may be generated from APP-CTFs produced from β-secretase (Belyaev et al., 2010). However, further investigation is needed to determine whether there is one or several AICD and what is the function of AICD. For instance, BACE-1 cleavage of APP and AICD derived from βCTF may contribute to learning, memory, and neuronal plasticity (Ma et al., 2007).

## Alzheimer’s Disease, Neurofibrillary Tangles, and Microtubule-Associated Tau

Neurofibrillary tangles are characterized by intraneuronal accumulation of fibrillar material named paired helical filaments (Kurt et al., 1997). Abnormally modified Tau proteins are the major components of this filamentous material. Tau proteins belong to the family of microtubule-associated proteins. A single gene, named *MAPT* located at position 17q21 encoded for several isoforms resulting from alternative splicing of exons 2, 3, and 10 in the human adult brain. There are six isoforms, half of which contains the exon 10 encoding sequence, two-third are having the exon 2 whereas the exon 3 is found in one-third of Tau isoforms and always in association with exon 2. The Tau isoforms differ from each other by the presence of either three (3R) or four repeat-regions (4R) in the carboxy-terminal (C-terminal) part of the molecule and the absence or presence of one or two inserts (29 or 58 amino acids) in the amino-terminal (N-terminal) part (Goedert et al., 1989a,b; Andreadis et al., 1992). Each of these isoforms is likely to have particular physiological roles since they are differentially expressed during development. For instance, only one Tau isoform, characterized by 3R and no N-terminal inserts, is present during fetal stages, while the six isoforms (with one or two N-terminal inserts and 3 or 4R) are expressed during adulthood (Kosik et al., 1989; Goedert and Jakes, 1990). Tau isoforms are differentially distributed in neuronal subpopulations or in yet underdetermined physiological conditions (Goedert et al., 1989a). However, in pathological conditions such as frontotemporal dementia linked to chromosome 17 or myotonic dystrophy, a mis-splicing of Tau is associated to the development of neurofibrillary degeneration (Vermersch et al., 1996; Hutton et al., 1998; Spillantini et al., 1998; Sergeant et al., 2001).

Tau proteins bind microtubules through repetitive regions in their C-terminal part. These repetitive regions are the repeat domains (R1–R4) encoded by exons 9 to 12 (Lee et al., 1989). The three (3R) or four copies (4R) are made of a highly conserved 18-amino acid repeat ending with a PGGG motif (Lee et al., 1988; Goedert et al., 1989b, Himmler et al., 1989; Lee et al., 1989) and separated from each other by less conserved 13- or 14-amino acid inter-repeat domains. Tau proteins are known to act as promoter of tubulin polymerization *in vitro*, and are involved in axonal transport (Weingarten et al., 1975; Cleveland et al., 1977; Brandt and Lee, 1993). Adult Tau isoforms with 4R (R1–R4) are more efficient at promoting microtubule assembly than the fetal isoform with 3R (R1, R3–R4; Goedert and Jakes, 1990; Butner and Kirschner, 1991; Gustke et al., 1994). Besides its major microtubule-binding, -stabilizing, paralleled-ordering functions, Tau also has other functions.

Tau proteins bind to spectrin and actin filaments (Selden and Pollard, 1983; Carlier et al., 1984; Correas et al., 1990; Henriquez et al., 1995). Through these interactions, Tau proteins may allow microtubules to interconnect with other cytoskeletal components such as neurofilaments (Miyata et al., 1986; Andreadis et al., 1995) and may restrict the flexibility of the microtubules (Matus, 1990). There is also evidence that Tau proteins interact with cytoplasmic organelles. Such interactions may allow for binding between microtubules and mitochondria (Jung et al., 1993). The Tau N-terminal projection domain also permits interactions with neural plasma membrane (Brandt et al., 1995). Thus, Tau may act as a mediator between microtubules and plasma membrane. This interaction has been defined as involving a binding between the proline-rich sequence in the N-terminal part of Tau proteins and the SH3 domains of Src-family non-receptor tyrosine kinases, such as Fyn. Studies have determined that human Tau Tyr18 and Tyr29 are phosphorylated by the Src-family tyrosine kinase Fyn (Williamson et al., 2002; Lee et al., 2004). Tau proteins was shown to co-sediment with lipid-raft fractions in response to Aβ and corresponded to phosphorylated Tau proteins at Tyr18 and Ser396/404 (Hernandez et al., 2009). In this latter study, it is suggested that Tau association to lipid-rafts may be necessary to mediate Aβ toxicity through the stabilization of Tau/Cdk5 interaction and thus suggesting that Tau as a potential signal transduction protein. The proline-rich region of Tau proteins is likely involved in the interaction with phospholipase C-γ (PLC-γ) isozymes (Hwang et al., 1996; Jenkins and Johnson, 1998). Hwang and colleagues have demonstrated *in vitro* that Tau proteins complex specifically with the SH3 domain of PLC-γ, and enhance its activity in the presence of unsaturated fatty acids such as arachidonic acid. These results suggest that in cells that express Tau proteins, receptors coupled to cytosolic phospholipase A2 may activate PLC-γ indirectly, in the absence of the usual tyrosine phosphorylation, through the hydrolysis of phosphatidylcholine to generate arachidonic acid (Hwang et al., 1996; Jenkins and Johnson, 1998). Altogether, these data indicate that Tau proteins may also play a role in the signal transduction pathway involving PLC-γ (for review see Rhee, 2001). In line with this idea, recent data demonstrate that Tau is necessary for glutamatergic signaling (Ittner et al., 2010). Overall, there is a growing body of evidence suggesting that tau may be close, interact, or even associate with intracellular vesicular compartment.

## The Spatiotemporal Brain Spreading of Neurofibrillary Degeneration

With aging, neurofibrillary tangles (NFT) spread from the transentorhinal cortex to the hippocampal formation. Neuropathological as well as biochemical assessment show that the Tau pathology spreads progressively, invariably, hierarchically, from the transentorhinal cortex to the whole neocortex, along cortico-cortical connections. The brain regions that are sequentially affected explain well the successive kind of cognitive impairments that characterize the disease: amnesia following the entorhinal and hippocampal degeneration: aphasia, apraxia, and agnosia with the involvement of the neocortex. Of course, amyloid and Tau pathology are present far early before the clinical symptoms (for review see Karran et al., 2011), because neuronal plasticity likely compensate at the first AD stages. Recently, the locus coeruleus has been described as the initiating region of NFD (Braak and Del Tredici, 2011). The Tau pathology is already distributed in the hippocampal formation and the temporal cortex at the “pre-clinical” stage of AD (Delacourte et al., 1999, 2002). Tau pathology, visualized as a triplet of abnormal Tau proteins, is systematically present in variable amounts in the entorhinal and hippocampal regions of non-demented patients aged over 75 years. When Tau pathology is found in other brain areas, it is always along a stereotyped, sequential pathway categorized into 10 stages according to the brain regions successively affected: transentorhinal cortex (S1), entorhinal (S2), hippocampus (S3), anterior temporal cortex (S4), inferior temporal cortex (S5), mid temporal cortex (S6), polymodal association areas (prefrontal, parietal inferior, temporal superior; S7), unimodal areas (S8), primary motor (S9a) or sensory (S9b, S9c) areas, and all neocortical areas (S10). Up to stage 6, the disease could be asymptomatic. In all of the cases at stage 7, individuals with two polymodal association areas affected by Tau pathology are cognitively impaired. This is of importance since it suggests that the spreading process occurs far before the occurrence of clinical symptoms and is also a very slow process likely transmitted through cortico-cortical connections therefore following rules and not randomly and most likely not diffusely. This hypothesis has been recently supported by different experimental works (Clavaguera et al., 2009, 2010; De Calignon et al., 2012; Liu et al., 2012). In contrast, amyloid deposits are diffusely progressing in the brain parenchyma (Braak and Braak, 1991; Duyckaerts and Hauw, 1997). However the mechanisms underlying the spreading and propagation of lesions remains poorly understood and current hypothesis, supported by the most recently published studies, suggested the spreading of AD lesions through interconnected neuronal circuitries. Among several hypotheses, we suggest that exosomes may contribute to this spreading process. Exosomes are produced from multivesicular endosomes and they will be first described followed by exosomes and the potential mechanism for the selectivity of spreading.

## Multivesicular Endosomes and Exosomes

**Figure 2:**
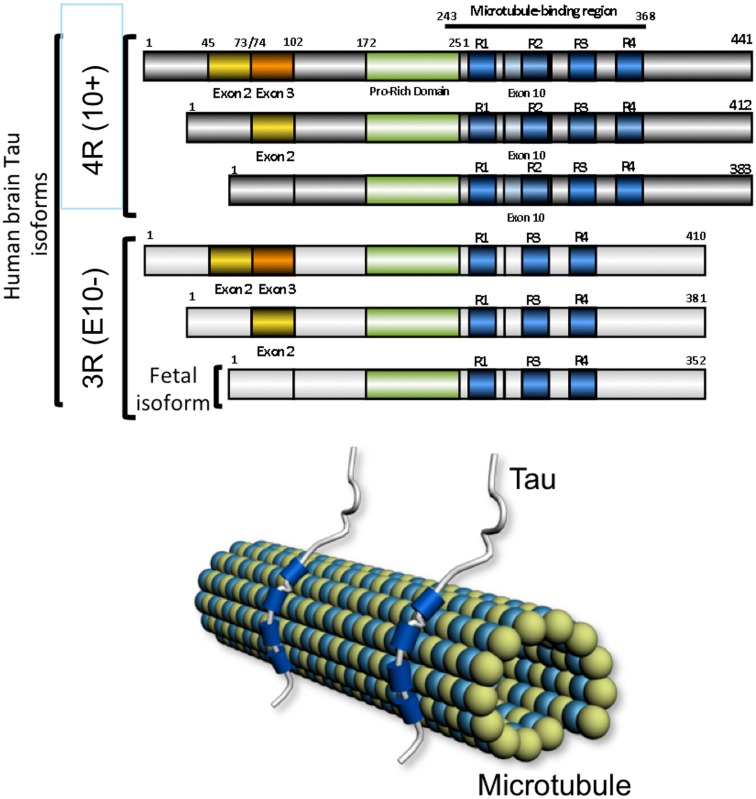
**The microtubule-associated protein *tau***. The six human brain isoforms of tau are represented. They differ by the inclusion of exclusion of exons 2 (yellow), exon 3 (orange), and exon 10 (light blue). The microtubule-associated domain are indicated in blue and depending on inclusion of exon 10 there are tau isoforms with 4 (4R) or 3 (3R) microtubule-binding regions. The fetal isoform is lacking the alternative encoding cassettes 2, 3, and 10. Tau protein binds the microtubule lattice through its microtubule-binding domains shown in blue.

Extracellular components, such as viruses, ligands, or diffusible factors and, part of the plasma membrane proteins are internalized during endocytosis. They are either recycled to the cell surface via early or recycling endosomes or, they are directed to late endosomes and finally delivered to lysosomes for degradation (for review see Gruenberg, 2009). Late endosomes are also known as multivesicular endosomes or MVBs (Gruenberg and Stenmark, 2004; Raposo and Marks, 2007; Rusten et al., 2011). They are required for the degradation of internalized material to fuse with lysosomes and are instrumental to several cellular functions including miRNA activity, mRNA transport, autophagy, cell polarity, receptor signaling, cytokinesis, and migration (Huotari and Helenius, 2011; Rusten et al., 2011). MVBs are large vesicles of several hundred nanometers that are characterized by numerous smaller intraluminal vesicles (ILVs) formed by the inward budding of the endosome limiting membrane. The formation of these ILVs requires sequential steps and the contribution of complex of multi-molecular machinery named Endosomal Sorting Complex Required for Transport (ESCRT). The ESCRT machinery is composed of four ESCRT protein complexes (0, I, II, and III) acting sequentially to sort ubiquitinated cargo and to form a coated subdomain on endosomes that forms the ILVs (Figure 2). Evidences for alternative pathways for cargos sorting into MVBs are emerging, which are independent of the ESCRT machinery but seems to depend on the lipid composition of raft-based micro domains. Proper cholesterol levels in late endosomes are required for normal MVBs formation and MVB-mediated membrane protein degradation (Kobuna et al., 2010). It was also shown that the phospholipid LBPA (lysobisphosphatidic acid) and ceramide possess the capacity to drive the formation of membrane invaginations (Matsuo et al., 2004; Trajkovic et al., 2008). Ubiquitination (Ub) is the main sorting signal for cargo entry into the vesicles that bud from the limiting membrane into the lumen of endosomes during the biogenesis of MVBs. A single Ub is sufficient to direct ILV targeting. Ub is recognized by an expanding cohort of endosomal proteins, which may act as Ub-sorting receptors responsible for binding and directing cargo toward ILVs like some ESCRT subunits, including VPS27/Hrs, VPS23/Tsg101, and VPS36/Eap45 (for review see: Piper and Katzmann, 2007). Many integral membrane proteins targeted for lysosomal degradation are ubiquitinated; however, non-ubiquitin sorting signals have also been described. Much less is known about non-Ub signals that sort proteins to ILVs; proteins which have been described to enter ILVs in an Ub-independent manner include Pmel17/Silver (Berson et al., 2003), TfR (Geminard et al., 2004), Nedd4 (Morita and Sundquist, 2004), Sna3 (McNatt et al., 2007; Oestreich et al., 2007). Two motifs “NTR” and “PKD” located on the extracellular part of Pmel17 are responsible for its targeting into the internal vesicles of MVBs (Theos et al., 2006) and COP9 signalosome (CSN)-associated protein Csn5 is involved in protein sorting into ILVs since siRNA of Csn5 causes a significant increase in both ubiquitinated and non-ubiquitinated proteins detected in exosomes (Liu et al., 2009).

Genetics also supports the importance of functional MVB in neurological disease and frontotemporal dementia. The gene encoding *CHMP2B* the ESCRT-III subunit was found to be mutated in a form of frontotemporal dementia (Skibinski et al., 2005) and amyotrophic lateral sclerosis (Cox et al., 2010) suggesting that functional MVBs are required to prevent accumulation of abnormal proteins that can disrupt neural function and ultimately lead to neurodegeneration (Filimonenko et al., 2007). Mutations in *CHMP2B* were first described in Danish and Belgian families but remain rare (Ghanim et al., 2010), yet accounting for less than 1% of Frontotemporal lobar degeneration linked to chromosome 3 (FTD-3; Isaacs et al., 2011; Gijselinck et al., 2012). Mutations CHMP2B^Intron5^ or CHMP2B^Delta10^ are supposed to lead to C-terminal truncation of CHMP2B. Brain tissue examination of patients with *CHMP2B* mutation showed enlarged vacuoles stained with a mannose-6-phosphate receptor antibody. The truncated protein impairs the fusion of endosome with lysosomes without obvious modification of protein sorting to MVB (Urwin et al., 2010). Ectopic expression of mutant CHMP2B^Intron5^ in primary cortical rodent neurons promote neuronal cell death through the failure of the mutant protein to dissociate from ESCRT-III complex. In parallel, an increased accumulation of autophagosomes was observed suggesting a defective fusion of autophagosomes with MVB (Lee et al., 2007). Staining of tissue from Alzheimer disease patients with CHMP2B showed an accumulation of the protein in vesicular structures resembling Granulo Vacuolar Degeneration (Yamazaki et al., 2010; Funk et al., 2011) suggestive of a defective autophagic and late endocytic pathways in AD and Frontotemporal lobar degeneration. MVB function is required for the proper clearance of intracellular protein aggregates such as TDP-43 or polyglutamine aggregates observed in Frontotemporal lobar degeneration and amyotrophic lateral sclerosis or Huntington disease, respectively (Filimonenko et al., 2007). Moreover, restoring or enhancing the lysosomal degradation and rates of autophagic protein turnover in a transgenic animal model of amyloid deposition can rescue the phenotype and decrease the amyloid burden (Yang et al., 2011). Together, a defective function of the endocytic pathway including MVB, autophagy, and lysosome may certainly contribute to the development of several neurodegenerative diseases including AD.

Alternatively to their fusion with lysosomes for degradation of their contents, MVBs have been described to fuse to the plasma membrane and release their content in the extracellular space (Harding et al., 1983; Pan et al., 1985), the ILVs contained in the MVBs when released are referred to as exosomes (Johnstone et al., 1987). Exosomes have a size ranging from 40 to 100 nm and can be secreted by many cell types including neuronal cells (Faure et al., 2006; Rajendran et al., 2006; Vingtdeux et al., 2007b; Lachenal et al., 2011). Exosomes are isolated from the media of cultured cells. However, purification of exosomes is not trivial since membrane fragments or cell debris can easily contaminate exosome preparations. Due to their small size, exosomes are obtained after filtration on 0.22 μm filters and by a series of centrifugation and sucrose gradient (Raposo et al., 1996; Wubbolts et al., 2003; Faure et al., 2006; Thery et al., 2006 for review see Olver and Vidal, 2007). Further immunoisolation can be used (Wubbolts et al., 2003). Several parameters should be evaluated to ascertain the purity of exosomes preparation. The first and likely most important characteristic is the observation of exosomes by transmission electron microscopy. Thus, exosomes have a typical cup-shape form. Several proteins are also common to exosomes and described in exosomes preparation that originate from different sources (for review Vella et al., 2008). Interestingly, several tetraspanins proteins are enriched in exosomes and may contribute to exosomes formation (Wubbolts et al., 2003; De Gassart et al., 2004). Tetraspanins are a growing family of transmembrane proteins with pleiotropic functions found associated with lipid-raft micro domains (for review Hemler, 2005). Interestingly, tetraspanins CD81 and CD9, which are found in exosomes derived from B-cells (Wubbolts et al., 2003), are co-purified with the γ-secretase interactome. Absence of those tetraspanins induces a partial disruption of γ-secretase activity or reduces γ-secretase substrate interaction (Wakabayashi et al., 2009). Although detailed molecular mechanisms remain unknown, together those results further support the idea that MVB and most possibly exosomes are important cellular compartments for APP metabolism regulation and that several γ-secretase regulators may act at this level.

MVBs fate can be affected by macroautophagy (hereafter referred to as autophagy). During autophagy, parts of the cytoplasm and organelles are encapsulated in double-membrane vacuoles called autophagosomes, which eventually fuse with lysosomes for degradation (for review see Levine et al., 2011). Under conditions that stimulate autophagy, MVBs are diverted to autophagic pathway with subsequent inhibition in exosomes secretion (Fader et al., 2008). Conversely, knockdown expression of ESCRT-I, -II, and -III proteins in cell models promotes the accumulation of autophagosomes or autolysosomes, suggesting that MVB and autophagy are intermingled and that loss of MVB function may promote autophagy as well as ineffective fusion of autophagosomes and lysosomes (for review see Rusten et al., 2011). Thus, loss of function of CHMP2B may impair both MVB and autophagosome maturation. With regards to Tau, the autophagy-lysosomal pathway contributes to the degradation of Tau (Wang et al., 2011). However, Tau protein inclusions are seldom detected in FTD-3 (Yancopoulou et al., 2003) and ubiquitin-positive inclusions are observed but TDP-43 negative (Holm et al., 2007).

**Figure 3:**
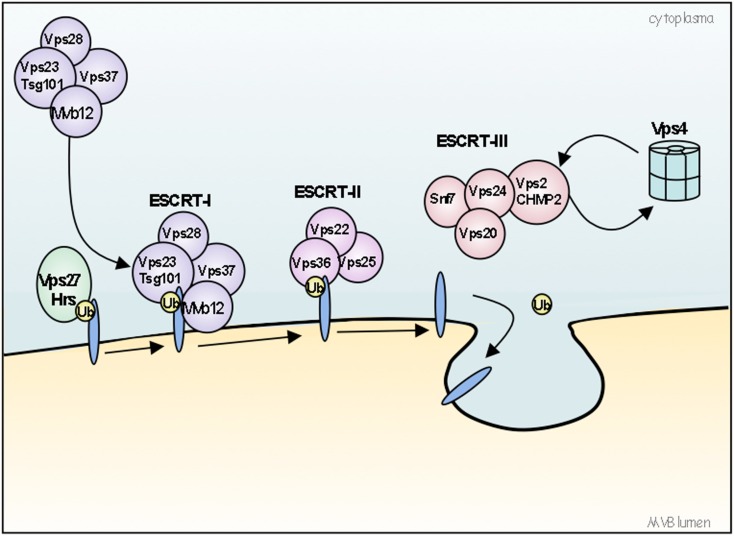
**Model for the ubiquitin-dependent sorting of proteins by the ESCRT machinery**. The ESCRT machinery is composed of four ESCRT protein complexes (0, I, II, and III) acting sequentially to sort ubiquitinated (ubiquitin is represented as Ub) cargo and to form a coated subdomain on endosomes that forms the ILVs. The VPS27/Hrs-Hse1/STAM complex (ESCRT-0) is first recruited to the endosomes by binding PI(3)P and ubiquitinated cargos. ESCRT-0 then recruits ESCRT-I (composed of Tsg101/VPS23-VPS28-VPS37) to the membrane, where ESCRT-I interacts with ubiquitinated cargos via its VPS23 subunit. Then, ESCRT-I recruits ESCRT-II complex (composed of VPS22/Eap30-VPS25/Eap25-VPS36/Eap45), which in turn initiates the oligomerization of ESCRT-III complex (composed of VPS2/CHMP2-VPS20/CHMP6-VPS24/CHMP3-Snf7/VPS32/CHMP4). ESCRT-I and II initiate the invagination of the limiting endosomal membrane. ESCRT-III deubiquitinating enzymes ensure the dissociation of ubiquitin residues from molecules before sequestration into MVBs. Finally, ESCRT-III recruits supplementary factors like Bro1 and Vsp4 AAA-ATPase. Bro1 will recruit a deubiquitination enzyme whereas VPS4 AAA-ATPase will work to break apart ESCRT-III and other ESCRT complexes, resulting in their dissociation from the membrane.

How exosomes are processed in recipient cells is not yet fully understood. Exosomes can be endocytosed into the endosomal system of recipient cells. Once internalized, exosomes could fuse with the limiting membrane of endosomes to deliver their cytoplasmic content into the host cell cytoplasm. It is also possible that exosomes could directly fuse with the plasma membrane. Although their exact function remains to be discovered, within the extracellular space and in biological fluids such as urine or serum, exosomes have been proposed to participate in different physiological and/or pathological processes such as neurodegenerative diseases (for review see Vella et al., 2008). They could be responsible not only for protein and lipids exchange between cells, but also for mRNA and microRNAs exchange (Valadi et al., 2007). Recently, miRNAs content of purified exosomes produced by dendritic cells were shown to ectopically repress target mRNAs of recipient dendritic cells (Montecalvo et al., 2011). Exosome release and content may be regulated by cellular stress. Thus DNA damage and activation of p53 induce the expression of protein that will be included inside exosomes (Yu et al., 2006). Exosomes may mediate a signal of cellular damage or stress. In the central nervous system, exosomes are proposed to constitute an intercellular communication system (for review see Mathivanan et al., 2011, 2012). Exosomes produced by glia-derived cells stimulate neurite outgrowth through a synapsin and NCAM dependent mechanism. In oxidative stress condition the synapsin released from exosomes is neuroprotective (Wang et al., 2011). AICD and several APP metabolites are found in exosomes derived from primary neuronal cultured cells (Vingtdeux et al., 2007b; Figure 3). L1 CAM that is also processed by γ-secretase (Riedle et al., 2009) is recovered in exosomes (Lachenal et al., 2011). Interestingly, modulators of the γ-secretase activity, such as inhibitors, modulate the release of APP-CTFs and Aβ associated to exosomes (Sharples et al., 2008). Altogether, there is a growing body of evidence suggesting that exosomes are small membrane-delineated cell-secreted material that may participate in cell-to-cell communication via both RNAs and proteins. Although speculative, if several intracellular domains of proteins processed by γ-secretase are internalized and secreted within exosomes, the fusion of those exosomes with surrounding cells may regulate gene expression by those intracellular domains and therefore constitute another cell communication system. However, if proteins that are degraded through late endosomes/lysosomes pathway are diverted from this normal degrading route, it may promote their accumulation, shape-transformation, and secretion via the multivesicular and exosome pathway.

## Prion-Like Propagation of Amyloid and Tau Pathologies

Besides being a potential system of intercellular communication, exosomes are also known to be instrumental to the dissemination of pathogens, whether those are viruses or proteinaceous pathogens. The first pathological protein described associated with exosomes was the prion protein (PrP; Fevrier et al., 2004; Alais et al., 2008). Prions diseases are fatal neurodegenerative disorders. They are associated with the conversion of the cellular prion protein (PrPc) into the scrapie PrP (PrPSc), an abnormal conformational state that tends to form amyloid deposits in brain tissue leading to dementia. Into its mis-folded conformation the PrPSc is thought to be infectious (for review see Aguzzi and Rajendran, 2009). Recent findings revealed an unexpected role for exosomes in dissemination of prions: exosomes from prion-infected neuronal cells have been demonstrated to be efficient initiators of prion propagation in uninfected recipient cells and, more importantly, to produce prion disease when inoculated into mice (Vella et al., 2007).

### Prion-like propagation of amyloid pathology

Exosomal release instead of lysosomal processing might be of advantage to cells having poor degradative capacities. In the context of AD, exosomes secretion could be a way to dispose of unwanted proteins. Indeed, maturation of autophagolysosomes and their retrograde transport are most possibly impeded in AD (Lee et al., 2011). The underlying mechanism behind the hypothesis that neurodegeneration in AD is triggered by protein spread, cell-to-cell, throughout brain areas could be the shipping of toxic agents such as Aβ or Tau by exosomes. What at the beginning would be beneficial (to bypass a degradation system which is overwhelmed) could become the reason why there is propagation of the disease thorough the brain. Aβ peptides are released by cells in association with exosomes (Rajendran et al., 2006) and interestingly, exosomal proteins such as Alix and flotillin-1 were observed around neuritic plaques, a lesion found in brains from AD patients (Rajendran et al., 2006) suggesting that exosomes-associated Aβ could be involved in plaque formation. MVBs are an intracellular compartment where internalized Aβ can grow into fibrils thereby MVBs may also contribute to amyloid plaque formation (Friedrich et al., 2010). Overall these results suggest that exosomes could play a role in the pathogenesis of AD. The idea of a “prion-like” propagation of Aβ lesions is also supported by results obtained *in vivo* in human wild-type APP transgenic mice (HuAPPwt) which do not develop Aβ deposits. Intracerebral inoculations of AD brain homogenates into the hippocampus of these mice lead to Aβ deposits which increased with age and spread to areas other than the site of injection (Morales et al., 2011). Propagation of Aβ-induced molecular impairments across synapses is also suggested in a transgenic animal model in which the expression of APP was restricted to the entorhinal cortex. With time and aging, amyloid deposits were observed in connected brain regions such as the dentate gyrus and CA1 pyramidal neurons (Harris et al., 2010). Entorhinal cortex neurons are neither directly connected to the granular cells of the dentate gyrus nor to CA1 pyramidal neurons. Therefore, amyloid deposits that appear with aging in those structures may originate from pathological Aβ species that propagate across synapses. A possible hypothesis would be that the pathological Aβ species are produced and released through the exosome pathway and exosomes are caught by interconnected neurons and trans-synaptically delivered to connected brain regions.

### Tau pathology spreading

The stereotype propagation scheme of neurofibrillary degeneration in AD is evidenced by neuropathological examination as well as biochemical analyses but until recently, hypotheses and experiments trying to address this question remained elusive. Neurofibrillary degeneration is following stereotypical brain circuitry following cortico-cortical connections therefore suggesting a loss of neurotrophic or surviving factor or a diffusible factor responsible for a cascade of molecular events leading to Tau aggregation and neuronal death. However, what is this propagating factor? What if Tau itself wouldn’t be the “missing link”? Thus, recent data suggest that neurofibrillary degeneration cortical spreading could follow a transmissible prion-like process. In fact, aggregates of PHF-Tau were purified from a transgenic mice model of neurofibrillary degeneration. Intracranial injection of this preparation was done in a different mouse model, which overexpresses human Tau protein but does not display Tau pathology. Following injection, the development of neurofibrillary degeneration was observed. This Tau pathology progressed from the injection site to neighboring brain structures, suggestive of a diffusible and transmissible propagating mechanism (Clavaguera et al., 2010). Very recently, Frost et al. (2009) have shown using a cell-based system that extracellular Tau aggregates are internalized inside cells and promote the mis-folding and fibrillization of Tau. Internalization of preformed Tau fibrils is facilitated by the use of a lipid-based protein delivery system (BioPorter^®^) and is likely mediated by endocytosis (Guo and Lee, 2011). The internalized preformed fibrils reduce microtubule-stabilization suggesting a loss-of-function of normal Tau in infected cells. Moreover, two recent studies showed that the tau pathology could spread *in vivo* through neuronal circuitry and trans-synaptic transmission (De Calignon et al., 2012; Liu et al., 2012). Although those transgenic models are valuable to decipher the molecular and cellular mechanisms of tau pathology spreading, the mechanism of tau protein conversion, oligomerization, secretion, trans-synaptic propagation remains elusive. There are some evidences suggesting that Tau may be secreted and secretion of Tau may differ depending on Tau isoform. Thus, Tau isoforms with exon 2 encoding sequence are likely not secreted and this exon 2 sequence is therefore suggested to repress Tau secretion (Kim et al., 2010). A good example of such a dilemma is fibroblast growth factor 2 that is a secreted growth factor without any signal peptide and that is also found in cell nucleus following its interaction with its cognate receptors (Meunier et al., 2009). Tau is likely secreted and is also located into the nucleus following stress conditions (Sultan et al., 2011). Tau secretion, as for Tau nuclear localization, may depend upon yet undefined conditions and therefore, contributions of MVB-exosomes pathways or autophagy-lysosomal pathways (Wang et al., 2009) remain completely open. Recent data strongly suggest that both pathways are possibly interconnected (Sahu et al., 2011). With regards to Tau, the degradation systems may bring insights for the potential routing of Tau to MVB-exosomes or autophagy-lysosome pathway. In NFT or more generally in aggregates, Tau is found ubiquitinated, thus suggesting that Tau may be processed by the proteasome (David et al., 2002). Ubiquitin-independent degradation system, such as caspase or calpain cleavage of Tau has also been described (Berry et al., 2003; Delobel et al., 2005; Ding et al., 2006; Carrettiero et al., 2009; Ferreira and Bigio, 2011). The autophagy-lysosomal pathway contributes to the degradation of Tau via the chaperone-mediated autophagy (CMA; Wang et al., 2009; for review see Wang et al., 2010). The CMA is a lysosome-mediated degradation system of cytosolic protein (for review see Arias and Cuervo, 2011). This system implies the recognition of substrates by a complex of chaperones and translocation of substrates inside lysosomes for further degradation. The CMA malfunction has been connected to the development of several neurodegenerative diseases including Parkinson disease and AD (Arias and Cuervo, 2011). Although speculative and purely hypothetic, through the use of CMA, aggregates of proteins or even oligomers could reach the lysosome and due to their low sensitivity to degradation (e.g., Tau aggregates), the fusion of lysosome with other vesicular structures such as MVB could finally lead to the release of aggregates outside the cell and contribute to their propagation following neuronal connections. Alternatively, proteins such as Tau would normally be addressed to lysosome by the CMA system but a defective lysosome could be the place where oligomers are generated and thereafter route to MVB/exosome pathway. More recently, two consecutive papers described the secretion of Tau protein by the exosome pathway (Saman et al., 2012; Simon et al., 2012). In MC1 neuroblastoma cells overexpressing the four-repeat Tau isoform with no N-terminal insert, a C-terminal truncated form or the full-length Tau protein was found co-purified with exosomes as well as associated with the exosome fraction obtained from human cerebrospinal fluid (Saman et al., 2012). Other Alzheimer associated markers are found in exosome such as Fyn-tyrosine kinase and Aβ (Segura et al., 2005; Rajendran et al., 2006). These proteins were also found in exosomes secreted by MC1 neuroblastoma cells (Saman et al., 2012). Interestingly, while the full-length Tau is recovered from COS cells overexpressing Tau, in HEK stably expressing Tau protein, a fragment encompassing Tau microtubule-binding repeat domains is principally found (Simon et al., 2012). However, endogenous Tau was not detected in exosomes derived from primary embryonic neuronal culture cells (Faure et al., 2006). Exosomes-associated secretion of Tau is only observed in overexpressing systems suggesting that the release of Tau by the exosomes pathway may contribute to eliminate the excess of intracellular Tau. Recent studies suggest that many transmissible pathogens such as PrP, α-synuclein, Huntingtin, Aβ are shuttle from a cell to another by secretion or tunneling nanotubes (for review see Goedert et al., 2010), however the mechanism of secretion or transmission and their regulation remain poorly understood.

### How tau pathology transmission is selective in sporadic tauopathies?

Conceptually, what mechanism can we propose to explain the prion-like spreading of Tau pathology affecting selective patterns of neurodegeneration and skipping nearby “less vulnerable” neuronal targets. That’s certainly a major fundamental question to address. Why in the scheme of spatiotemporal spreading and propagation of lesions in AD and other sporadic Tauopathies such as Pick’s disease, progressive supranuclear palsy or corticobasal degeneration only selective neuronal subpopulations are affected (for review see Sergeant et al., 2008). As for instance, affected neurons in AD essentially belong to the cholinergic system whereas those degenerating in PSP are dopamine neurons (Murphy et al., 2008). One possibility would be that selectivity of propagation could follow neuronal circuitry through synaptic transmission. This would be possible if exosomes were preferentially released at the synaptic junction, as suggested by Smalheiser (2007). There are emerging evidence that exosomes are produced and secreted by neurons and that synaptic activity could enhance exosomes secretion (Lachenal et al., 2011). However, the demonstration derives from *in vitro* experiments using primary neuronal embryonic culture cells. Study of exosomes in tissue yet remains highly challenging although recently it was shown that exosomes could be detected in synaptic boutons at the *Drosophila* larval neuromuscular junction (Koles et al., 2012). Consequently, little if not nothing is known about the neuronal localization, regulation of release and their propensity of diffusion *in vivo*.

There are therefore other possibilities, such as the tunneling nanotubes (for review see Goedert et al., 2010). Tunneling nanotubes are fine membrane channels that have recently been described in mammalian cells for communication between cells but also for cell-to-cell propagation of mis-folded PrPs (for review see Gerdes et al., 2007; Gousset et al., 2009; Zhang, 2011). These tunneling nanotubes could also propagate other transmissible mis-folded proteins such as Aβ (Zhang, 2011) but the question of selectivity of transmission remains open. However, it is note of worthy that tunneling nanotubes is induced following oxidative stress in rodent hippocampal neurons and astrocytes. Cell-to-cell connection and communication of intracellular organelles or Aβ could be trigged by cellular stress (Zhang, 2011). Following this scheme, the stressed cell, such as a degenerating neuron, would connect via tunneling nanotubes to closely surrounding or connected neurons to deliver the pathogenic protein. However hypothetic, tunneling nanotubes is an emerging mechanism of cell communication under stress conditions that may or could contribute to neurodegenerative diseases (Goedert et al., 2010; Zhang, 2011).

Coming back to exosomes and now considering that exosome release and secretion is controlled and localized to pre- or post-synaptic locations then several hypotheses can be postulated. In both pre- and post-synaptic situations propagation through exosomes would be closely dependent upon neuronal connections, as far as the diffusion of exosomes is following a paracrine or “juxtacrine” rule of diffusion (Mathivanan et al., 2011). Thus, only interconnected neurons would disseminate toxic species via exosomes. We can also hypothesize that exosomes originating from different type of neurons (e.g., cholinergic, GABAergic, glutamatergic, dopaminergic…) may contain specific membrane-associated biomarkers. Intercellular communication mediated by exosomes may result from passive fusion of exosome membrane with the plasma membrane of the targeted cell or may use a ligand receptor system. In line with the latter system, the selectivity of intercellular communication could result from specific interaction between ligand and receptor. There are several examples that could illustrate a selectivity of propagation of exosomes using this ligand receptor selectivity. For instance, protocadherin is a cluster of 52 cadherin-like genes with a singular organization. The amino-terminal region of protocadherins is encoded by three sets of separate exons arranged in three clusters (alpha, beta, and gamma). N-terminal encoding exons are spliced with one of three carboxy-terminal encoding exons. Alternative splicing generates an extraordinary diversity of protocadherin isoforms suggested to confer selective and specific intermolecular membrane-associated protein interactions (Wu and Maniatis, 2000; Wang et al., 2002). The second example is DSCAM, the *Drosophila* homolog of human Down syndrome cell adhesion molecule that belongs to the axonal guidance receptor family. Alternative splicing of DSCAM can generate as many as 38016 mRNA isoforms and therefore lead to expression of huge protein diversity (Schmucker et al., 2000). More interestingly, one DSCAM protein isoform binds exactly to the same isoform but not a slightly different one, making the binding of DSCAM isoforms very stringent (Wojtowicz et al., 2004). As for DSCAM, the selectivity of transmission pattern could be mediated following an axonal guidance-like system. In a very simplified view, axonal guidance is driven by equilibrium between attractive and repulsive signals through specific signaling pathways, allowing axonal growth and connection to its specific neuronal target (for review see Bashaw and Klein, 2010). Thus exosome release from one type of cell will be attracted by its target cell and repulsed by surrounding cells. Altogether, examples provided could contribute to neuronal communication and propagation of mis-folded proteins along specific identified neuronal circuitries. Although all these hypotheses could be envisioned a better knowledge of the metabolism of exosomes *in vitro* and *in vivo* is necessary to address this problematic.

## Conclusion

**Figure 4:**
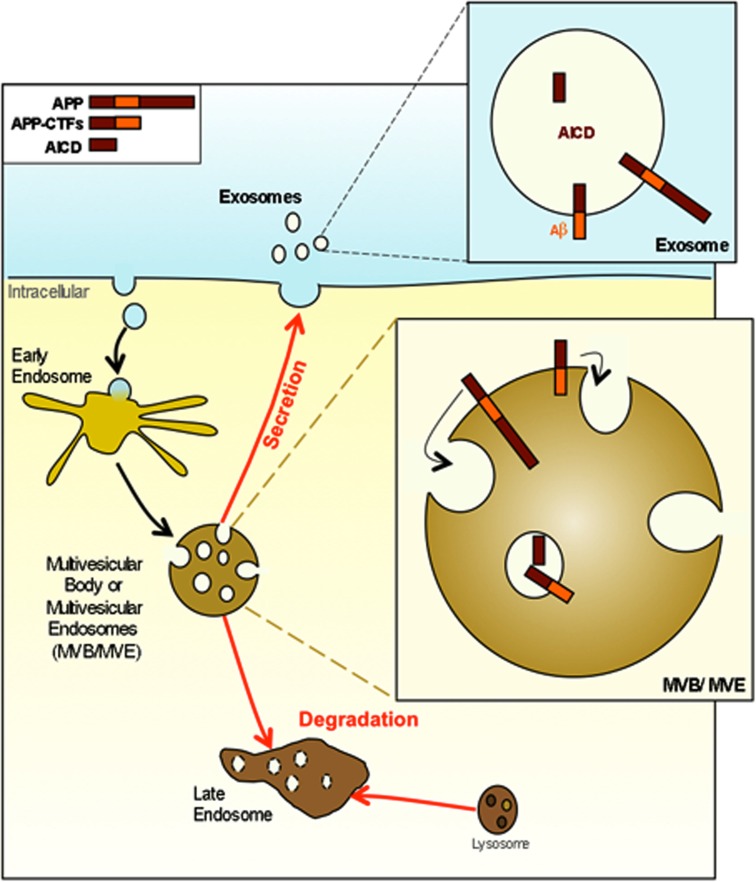
***APP* and its metabolites are present in multivesicular bodies and exosomes**. APP and APP-CTFs are internalized and directed into the internal vesicles of multivesicular bodies (MVB). At this point APP and its metabolites can either be degraded after the fusion of MVB with lysosomes or can be released in the extracellular space in association with exosomes consecutively to the fusion of MVB with the plasma membrane.

**Figure 5:**
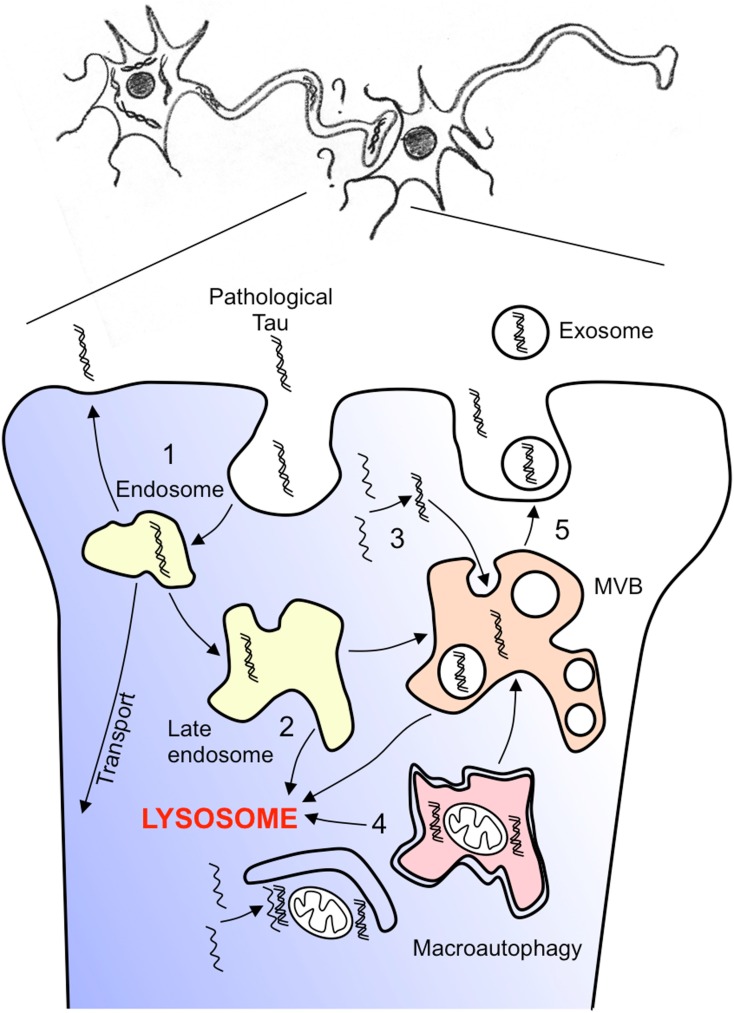
**Hypothesis of pathological tau spreading**. How can pathological tau species spreads through trans-synaptic connections? A non-exhaustive representation of several hypotheses are given. (1) Pathological species can be endocytosed recycled, amplified, transport, and secreted (2) Endocytosed species can follow the endosome lysosome routing and either be addressed to lysosomes or to multivesicular endosomes/bodies (3) Pathological tau species that are produced in the cell soma are included into multivesicular bodies by inward budding of the late endosome membrane (4) Pathological tau species can be included into large autophagic vesicles by macroautophagy and further fused with multivesicular bodies (5) pathological tau species can be release by fusion of multivesicular bodies with the plasma membrane at the synaptic junction and capture by the connected dendrite.

Among pathophysiological mechanisms of neurodegenerative diseases leading to intra or extracellular protein aggregates, a consensual mechanism support a prion-like propagation of mis-folded proteins. However, when this mechanism implies the propagation from cell-to-cell, shuttling pathways incriminated remains poorly understood (see Figures 4 and 5 for potential hypothesis). A growing body of evidence suggests that the endocytic – multivesicular endosome and exosome pathways may contribute to this process and to the development of several neurodegenerative diseases. Much is known about the routing of proteins through those recycling or degradative pathway but much less is known about the contribution of those systems to the development of neurodegenerative diseases. However, this MVB – exosome system can be diverted from its physiological function as for instance to produce human immunodeficiency viral particles (Nguyen et al., 2003; for review see Gould et al., 2003). Following this hypothesis, the autophagy-lysosome and/or MVB – exosome pathways could also be diverted to deliver and propagate toxic oligomers or aggregates in neurodegenerative diseases such as AD. Blocking the diffusion of those toxic and mis-folded species by this secretory pathway could also represent a potential therapeutic approach of neurodegenerative diseases such as Amyloidopathies, Tauopathies, Synucleinopathies, or more largely Foldopathies all of which are likely sharing a “prion-like” propagation of toxic mis-folded proteins.

## Conflict of Interest Statement

The authors declare that the research was conducted in the absence of any commercial or financial relationships that could be construed as a potential conflict of interest.
